# Motor co-activation in siblings of patients with juvenile myoclonic epilepsy: an imaging endophenotype?

**DOI:** 10.1093/brain/awu175

**Published:** 2014-07-07

**Authors:** Britta Wandschneider, Maria Centeno, Christian Vollmar, Mark Symms, Pamela J. Thompson, John S. Duncan, Matthias J. Koepp

**Affiliations:** 1 Department of Clinical and Experimental Epilepsy, UCL Institute of Neurology, Queen Square, London WC1N 3BG, UK; 2 Imaging and Biophysics Department, UCL Institute of Child Health, 30 Guilford Street, London WC1N 1EH, UK; 3 Department of Neurology, Ludwig-Maximilians-Universität, Marchioninistr. 15, 81377 Munich, Germany

**Keywords:** juvenile myoclonic epilepsy, functional magnetic resonance imaging, endophenotype, genetic, motor system

## Abstract

Juvenile myoclonic epilepsy is a heritable idiopathic generalized epilepsy syndrome, characterized by myoclonic jerks and frequently triggered by cognitive effort. Impairment of frontal lobe cognitive functions has been reported in patients with juvenile myoclonic epilepsy and their unaffected siblings. In a recent functional magnetic resonance imaging study we reported abnormal co-activation of the motor cortex and increased functional connectivity between the motor system and prefrontal cognitive networks during a working memory paradigm, providing an underlying mechanism for cognitively triggered jerks. In this study, we used the same task in 15 unaffected siblings (10 female; age range 18–65 years, median 40) of 11 of those patients with juvenile myoclonic epilepsy (six female; age range 22–54 years, median 35) and compared functional magnetic resonance imaging activations with 20 age- and gender-matched healthy control subjects (12 female; age range 23–46 years, median 30.5). Unaffected siblings showed abnormal primary motor cortex and supplementary motor area co-activation with increasing cognitive load, as well as increased task-related functional connectivity between motor and prefrontal cognitive networks, with a similar pattern to patients (*P* < 0.001 uncorrected; 20-voxel threshold extent). This finding in unaffected siblings suggests that altered motor system activation and functional connectivity is not medication- or seizure-related, but represents a potential underlying mechanism for impairment of frontal lobe functions in both patients and siblings, and so constitutes an endophenotype of juvenile myoclonic epilepsy.

## Abstract (precis)

In juvenile myoclonic epilepsy (JME), myoclonic jerks are often triggered by cognitive effort. Wandschneider *et al.* report co-activation of the motor and prefrontal cognitive networks in unaffected siblings, similar to that previously reported in patients themselves. This co-activation could constitute a heritable marker for further genetic studies of JME.

## Introduction

Juvenile myoclonic epilepsy is a common idiopathic epilepsy syndrome (Zifkin *et al.*, 2005; Berg and Millichap, 2013; Delgado-Escueta *et al.*, 2013), characterized by symmetric, myoclonic jerks, mostly affecting upper limbs, generalized tonic-clonic seizures and, more rarely, absence seizures (Janz, 1985; Kasteleijn-Nolst Trenite *et al.*, 2013). A complex polygenetic aetiology is suspected in most cases (Delgado-Escueta *et al.*, 2013) and clinical genetic studies support a high genetic predisposition: first-degree relatives have an increased risk for epilepsy with up to 6% affected, mostly with idiopathic generalized epilepsy syndromes (Janz *et al.*, 1989; Vijai *et al.*, 2003). Reports on high syndrome concordance amongst first-degree relatives of 30% (Marini *et al.*, 2004) and very high monozygous concordance reported by twin studies support a major heritable disease component (Vadlamudi *et al.*, 2004; Corey *et al.*, 2011).

Reflex mechanisms of seizure precipitation are common in juvenile myoclonic epilepsy, including photic stimulation but also cognitively triggered jerks, by reading, decision-making or planned movement, leading to jerking of the body part that is engaged in task execution, usually the hand (Inoue *et al.*, 1994; Inoue and Kubota, 2000; Matsuoka *et al.*, 2000, 2005; Guaranha *et al.*, 2009).

Neurobehavioural findings of impaired working memory and executive functions (Devinsky *et al.*, 1997; Sonmez *et al.*, 2004; Wandschneider *et al.*, 2012) corroborated evidence from advanced imaging studies for subtle structural and functional changes within the dorsolateral prefrontal and medial frontal lobes and thalamo-fronto-cortical pathways (Koepp *et al.*, 1997; Savic *et al.*, 2004; Pulsipher *et al.*, 2009; O'Muircheartaigh *et al.*, 2011*,* 2012). In a previous study (Vollmar *et al.*, 2011, 2012), we investigated the interaction of motor and cognitive networks in juvenile myoclonic epilepsy using an n-back functional MRI task, which assesses visual-spatial working memory with increasing cognitive demand and also entails a complex motor component (Kumari *et al.*, 2009). Patients with juvenile myoclonic epilepsy showed an abnormal motor cortex co-activation with increasing task demand during the working memory task. In addition, both functional and structural connectivity were increased between cortical motor areas and dorsolateral prefrontal cognitive networks, and decreased within prefrontal cognitive networks (presupplementary motor area to frontopolar regions), providing a potential underlying mechanism for both cognitively triggered jerks and cognitive impairment in juvenile myoclonic epilepsy. Although we observed a ‘normalization’ of this altered co-activation with increasing doses of the anti-epileptic drug sodium valproate, our previous study could not disentangle whether motor system hyperconnectivity to cognitive networks is a disease-underlying mechanism or a consequence of seizures and/or treatment.

Because juvenile myoclonic epilepsy has a high heritability and neurobehavioural studies in unaffected siblings have described traits of its broader phenotype, such as frontal lobe cognitive impairment (Wandschneider *et al.*, 2010), we sought to investigate whether motor system co-activation during a working memory task is an endophenotype of juvenile myoclonic epilepsy. Endophenotypes are manifest in an individual whether or not the condition is active, are heritable and are found more frequently in non-affected family members of diseased individuals than in the general population (Gottesman and Gould, 2003). As the genetic risk for epilepsy is higher for siblings of patients with juvenile myoclonic epilepsy than their offspring or parents and syndrome traits have also been more frequently reported in siblings than in other first-degree relatives, this study focused on investigating unaffected siblings (Janz *et al.*, 1989; Jayalakshmi *et al.*, 2006). Index patients and siblings are also more likely to be comparable for age, upbringing and socioeconomic background than patients and other first-degree relatives. Specifically, we hypothesized that unaffected siblings of patients with juvenile myoclonic epilepsy will show (i) abnormal functional MRI activation patterns compared with healthy control subjects in previously defined regions of interest in the motor cortex of patients with juvenile myoclonic epilepsy; and (ii) increased functional connectivity between the motor system and frontoparietal cognitive networks.

## Materials and methods

This study was approved by the Research Ethics Committee of the University College London Institute of Neurology and University College London Hospitals. Written informed consent was obtained from all study participants.

### Participants

Fifteen unaffected siblings of 11 patients with juvenile myoclonic epilepsy participated after contact with the consent of the related juvenile myoclonic epilepsy index patient. Patients with juvenile myoclonic epilepsy were either identified from a previous functional MRI study (*n* = 5) (Vollmar *et al.*, 2011) or recruited from epilepsy outpatient clinics at University College London Hospitals (*n* = 6). Twenty healthy control subjects were also included [siblings/patients/controls: 10/6/12 females; age: siblings: median 40 (interquartile range, IQR: 21) years; patients: 35 (23); controls: 30.5 (7)]. Siblings and controls were comparable for age (Mann-Whitney U = 98.500, *P* = 0.086), gender (Pearson χ^2^ *P* = 0.686) and IQ (Table 1).

**Table 1:** Neuropsychological test results

**Cognitive measures**	**Controls**	**Siblings**	Statistical analysis*	
	**Median (IQR)**	**Median (IQR)**	**U**	***P***
IQ				
NART	110 (10)	107 (19)	71.500	0.574
WAIS-III (raw scores)				
Verbal comprehension				
Vocabulary	50 (21)	49 (24)	73.000	0.935
Similarities	26.5 (12)	24 (5)	51.000	0.196
Working memory				
Digit Span	17 (6)	20 (5)	43.000	0.080
Arithmetics	14 (10)	14 (7)	68.000	0.862
Expressive Language				
Graded Naming Test	23 (5)	23 (7)	64.500	0.567
Verbal Learning				
List Learning (AMIPB) (Trials 1–5)	56 (13)	56 (6)	46.000	0.215
Non-verbal Learning				
Design Learning (AMIPB) (Trials 1–5)	40 (11)	36 (11)	60.500	0.152
Psychomotor speed				
Trail Making Test A (s)	25 (15)	25 (13)	66.000	0.413
Mental flexibility				
Trail Making Test time B − A (s)	19 (13)	23 (18)	49.500	0.235
Verbal fluency				
Categorical fluency	18 (3)	18 (5)	60.000	0.808
Letter fluency	14 (5)	14 (4)	82.000	0.862

*The Mann-Whitney U-Test was applied for behavioural measures. All variables are reported as raw items, except for Trail Making Test (time in seconds) and verbal IQ points. AMIPB = Adult Memory and Information Processing Battery; NART = National Adult Reading Test; WAIS = Wechsler Adult Intelligence Scale.

All index patients had a typical history of juvenile myoclonic epilepsy with myoclonic jerks, generalized tonic-clonic seizures and, in some, absence seizures. Disease onset was in adolescence, EEGs showed generalized polyspike wave complexes and clinical MRI were normal.

Three patients reported movement-related jerks in the active hand: one when playing the guitar and writing down musical notes simultaneously; one when playing the violin or touch-typing on a screen; and one patient reported jerks during tasks requiring fine motor skills.

No sibling had ever experienced seizures, except for one who had suffered two clearly provoked (sleep deprivation) generalized tonic-clonic seizures over 20 years before study participation, without any further seizures, and without anti-epileptic medication.

In five families with juvenile myoclonic epilepsy, other relatives apart from the index patient suffered from epilepsy. There was a family history of febrile convulsions in two cases. Healthy controls had no history of epilepsy or other neurological disease and no family history of epilepsy.

### MRI data acquisition

MRI data were acquired on a GE Excite HDx 3 T scanner (General Electric Medical Systems) with a multichannel head coil. A 50-slice gradient echo planar imaging sequence was used in axial orientation with 2.4-mm thickness and 0.1-mm gap providing full brain coverage. Slices had a 64 × 64 matrix, voxel size was 3.75 × 3.75 mm. Repetition time was 2500 ms, echo time was 25 ms.

### Functional MRI working memory paradigm

Participants were scanned with the same working memory paradigm as used previously (Vollmar *et al.*, 2011, 2012). An adaptation of the visual-spatial n-back working memory task was employed (Kumari *et al.*, 2009). Dots were presented randomly in four possible locations on a screen. Participants responded by moving a joystick with their right hand. They monitored the locations of dots and had to move the joystick to the position of the currently presented dot in the ‘0 Back’ condition or to the position of the dot in the previous presentation (‘1 Back’) or two (‘2 Back’) presentations earlier. Each condition lasted 30 s, was repeated five times in a pseudorandom order and alternated with rest blocks of 15 s. During the total duration of the paradigm (11 min 20 s), 272 echo planar imaging volumes were acquired.

### Functional MRI processing and analysis

Functional MRI data were analysed with Statistical Parametric Mapping 8 (www.fil.ion.ucl.ac.uk/spm). Images were realigned, normalized to an acquisition-specific echo planar imaging template in Montreal Neurological Institute space, resampled to isotropic 3 × 3 × 3 voxels and smoothed with an 8 × 8 × 8 mm kernel.

Single subject statistical analysis was carried out applying a full factorial block design. Movement parameters were entered as regressors of no interest. Task conditions were modelled separately as 30-s blocks and convoluted with the Statistical Parametric Mapping canonical haemodynamic response function. For each subject, contrasts were defined by comparing task conditions against rest and comparing task conditions with working memory load (‘1 Back’ and ‘2 Back’) against the control task (‘0 Back’). Hence by controlling for motor response and visual attention, only cortical activation due to the working memory load was revealed.

At the second level, group comparisons were carried out using two-sample *t*-tests or a full factorial design. The level of significance was set at *P* < 0.001 uncorrected with an extent threshold with minimum cluster size of 20 voxels (Lieberman and Cunningham, 2009). Where appropriate, performance during the n-back task was entered as a regressor of no interest. Functional MRI results were rendered on a 3D surface previously created from the Montreal Neurological Institute_152_T1 data set (Vollmar *et al.*, 2011).

### Functional connectivity

An independent component analysis was carried out using MELODIC from the FMRIB software library (FSL, http://www.fmrib.ox.ac.uk/fsl/) to identify different network components.

A 4D file of the realigned, normalized and smoothed images was created for each subject. Image data were prefiltered with a high-pass filter with a cut-off at 100 s. The algorithm was restricted to identify 32 components common across all subjects. Motor and working memory components were visually identified at the group level. Individual time series for each component were extracted for each subject using Dual Regression (Filippini *et al.*, 2009).

Subsequently, for each subject and each component, connectivity maps were generated by regressing the time series in a general linear model including movement parameters as regressor of no interest. Group comparisons were carried out with two-sample *t*-tests or a full factorial design.

### Behavioural data and statistical analysis

All participants underwent a standardized neuropsychological assessment. The Nelson Adult Reading Test was used as an index of intellectual level (Nelson, 1982). The Vocabulary and Similarities subtests from the Wechsler Adult Intelligence Scale III were used to measure verbal comprehension and the Digit Span and Mental Arithmetic subtests from the same scale provided a measure of working memory. Expressive language functions were measured using the Graded Naming Test (McKenna and Warrington, 1983). The List Learning and Design Learning Subtests from the Adult Memory and Information Processing Battery measured verbal and visual learning, respectively (Baxendale *et al.*, 2008). The Trail Making Test provided a measure of psychomotor speed (Trail Making A) and mental flexibility (Trail Making B-A). Participants also completed measures of letter and category fluency.

Behavioural and clinical data were analysed using SPSS Statistics Version 20.0 (IBM). Mann-Whitney U Test was applied to non-parametric data and Chi-square tests to categorical data. The level of significance was set at *P* < 0.05.

## Results

### Behavioural performance on standardized neuropsychology testing and during the functional MRI working memory task

There were no significant group differences in performance on the neuropsychological test battery. The results are detailed in Table 1.

Both, siblings and healthy controls performed equally well during the ‘0 Back’ condition [success rate median (IQR) siblings: 95 (10)%; controls: 93 (11); Mann-Whitney U = 143.000, *P* = 0.831). However, siblings performed worse in the ‘1 Back’ [siblings: 77 (43), controls: 92.5 (11.75); Mann-Whitney U = 82.000 *P* = 0.023] and ‘2 Back’ condition [siblings: 55 (41), controls: 88 (31.5); Mann-Whitney U = 69.500, *P* = 0.006]. Performance measures were therefore entered as regressors of no interest in the functional MRI group comparisons.

### Effects of increasing cognitive load on functional MRI activations and de-activations

**Figure 1:**
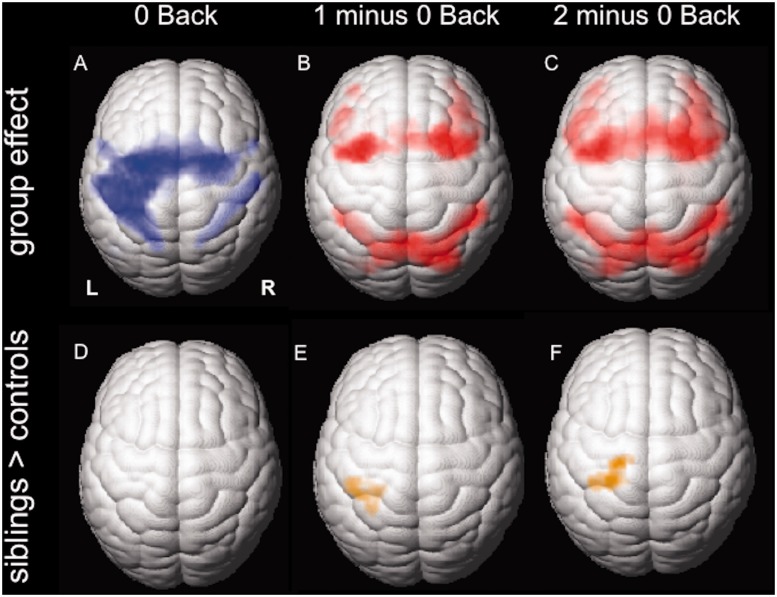
Group functional MRI activation from working memory and group differences. Group functional MRI activation maps from juvenile myoclonic epilepsy siblings and healthy controls. (**A–C**) Cortical activation for the three different task conditions: motor cortex and supplementary motor area for ‘0 Back’ (**A**), bilateral frontal and parietal activation for ‘1 minus 0 Back’ and ‘2 minus 0 Back’ (**B** and **C**). (**D–F**) Activation patterns in unaffected juvenile myoclonic epilepsy siblings compared to controls [inclusively masked for task-dependent deactivation maps of healthy controls (*P* < 0.001 uncorrected; 20 voxel threshold extent)]: no difference for the ‘0 Back’ condition (**D**), but attenuated deactivation in the motor cortex (**E**) and the supplementary motor area (**F**) with increasing task demand in the working memory contrasts was seen.

In the ‘0 Back’ condition, due to the right hand motor response, all subjects showed a left central and bilateral supplementary motor area activation (Fig. 1A). By controlling for motor response and subtracting ‘0 Back’ from ‘1 Back’ and ‘2 Back’, cortical activations due to working memory were isolated. All participants showed significant bilateral prefrontal and parietal working memory network activation (Fig. 1B and C). Group differences are shown in Fig. 1D–F. There were no group differences detectable during the ‘0 Back’ condition. However, in the ‘1 minus 0 Back’ contrast, there was a significant difference in activation patterns between siblings compared to controls within the region of interest, the motor cortex. The effect became more prominent and extended to the supplementary motor area with increasing cognitive demand in the ‘2 minus 0 Back’ contrast.

To disentangle whether the differences between siblings and controls observed were due to an increase of the task-positive network or an impaired deactivation of the task-negative network in siblings relative to controls, we masked the results either by group effects of the task-positive (‘2 minus 0 Back’) or task-negative network (‘0 minus 2 Back’) for controls (Fig. 1E and F). Areas of difference corresponded to the task-negative network in controls. Hence the effect observed in the motor system in siblings is due to impaired deactivation of this area with increasing working memory load. There were no areas of greater activation in controls compared to siblings.

**Figure 2:**
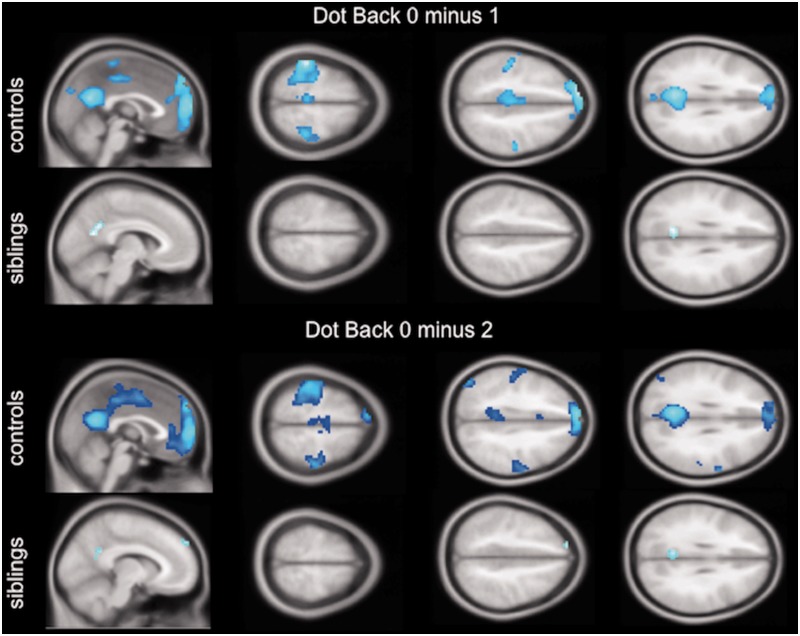
Group effect of task-dependent deactivation in controls and siblings for the two negative working memory contrasts (‘Dot Back 0 minus 1’, ‘Dot Back 0 minus 2’). In controls, the task negative contrast shows bilateral deactivation of the motor cortex and supplementary motor area with increasing task demand, as well as deactivation of the precuneus and medial prefrontal areas (default mode network). Less group deactivation effects in these areas are observed in siblings (*P* < 0.001 uncorrected; 20 voxel threshold extent).

To further explore this effect, group maps of the task negative network are displayed in Fig. 2. Whereas controls deactivate the primary motor cortices with increasing cognitive task demand, as well as areas in the default mode network, i.e. precuneus and medial frontal and orbitofrontal areas, the group effect in siblings shows less deactivation in these areas.

### Task performance and functional MRI results

**Figure 3:**
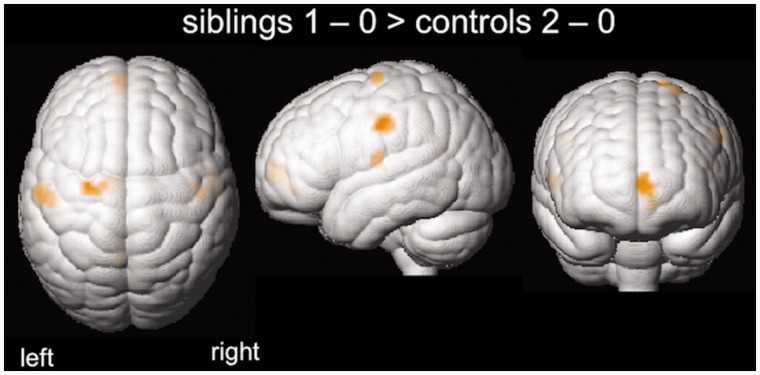
*Post hoc* group comparisons of functional MRI activation patterns during comparable working memory task performance. Siblings’ performance accuracy during the ‘1 Dot Back’ was comparable with controls’ performance during ‘2 Dot Back’ condition. There was attenuated deactivation in the lateral primary motor cortex bilaterally and left supplementary motor area, as well as in the left medial prefrontal cortex for ‘1 minus 0 Back’ in siblings compared to ‘2 minus 0 Back’ in controls (*P* < 0.005 uncorrected; 20 voxel threshold extent; inclusively masked for areas of task-related deactivation in controls). There were no areas of higher activation in controls.

To control for performance effects, in addition to treating performance as a confounder of no interest, we performed a *post hoc* group comparison between contrasts ‘1 minus 0 Back’ in siblings and ‘2 minus 0 Back’ in controls, since controls’ performance accuracy in the ‘2 Back’ condition was comparable to siblings’ accuracy in the ‘1 Back’ condition [success rate median (IQR) siblings ‘1 Back’: 77 (43) %; controls ‘2 Back’: 88 (31.5); Mann-Whitney U = 121.000, *P* = 0.347] (Fig. 3). As in the previous analysis, siblings show an attenuated deactivation of the motor areas and parts of the default mode network. There were no areas of greater activation in controls compared to siblings.

### Comparison of functional MRI results in patients with juvenile myoclonic epilepsy, siblings and healthy controls

We entered contrast images for ‘1 minus 0 Back’ and ‘2 minus 0 Back’ of siblings, controls and the 11 juvenile myoclonic epilepsy index patients in a full-factorial design with group as factor. Performance accuracy was different between the three groups for the ‘2 Back’ performance (Kruskall-Wallis Test: ‘0 Back’ *χ^2^* = 0.337, *P* = 0.845; ‘1 Back’ *χ^2^* = 5.757, *P* = 0.056; ‘2 Back’ *χ^2^* = 8.178, *P* = 0.017). *Post hoc* group comparisons showed that these performance differences were due to siblings performing worse than controls, with performance accuracy of patients with juvenile myoclonic epilepsy being comparable to controls’ and siblings’ ‘2 Back’ performance (patients versus controls: Mann-Whitney U = 72.000, *P* = 0.123; patients versus siblings: Mann-Whitney U = 56.500, *P* = 0.180).

**Figure 4:**
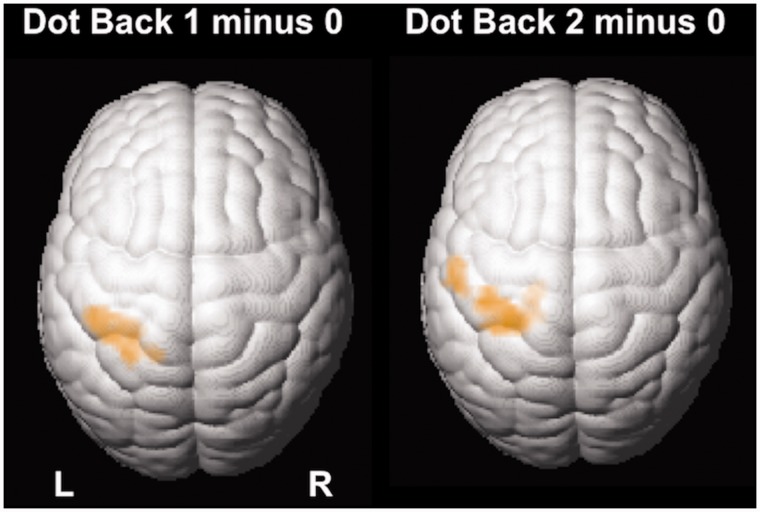
Conjunction analysis. In a conjunction analysis of patients greater than controls and siblings greater than controls for ‘Dot Back 1 minus 0’ and ‘Dot Back 2 minus 0’, patients and their siblings share significant areas of co-activation in the left motor cortex when compared to controls (conjunction, *P* < 0.005 uncorrected; 20 voxels threshold extent).

Performance scores were entered as regressors of no interest. There were no differences in activations between patients with juvenile myoclonic epilepsy and siblings for either working memory contrasts (not shown). In a conjunction analysis of areas activating in both patients with juvenile myoclonic epilepsy and siblings more than controls, we identified common areas of significant activations in the left primary motor cortex (Fig. 4).

**Figure 5:**
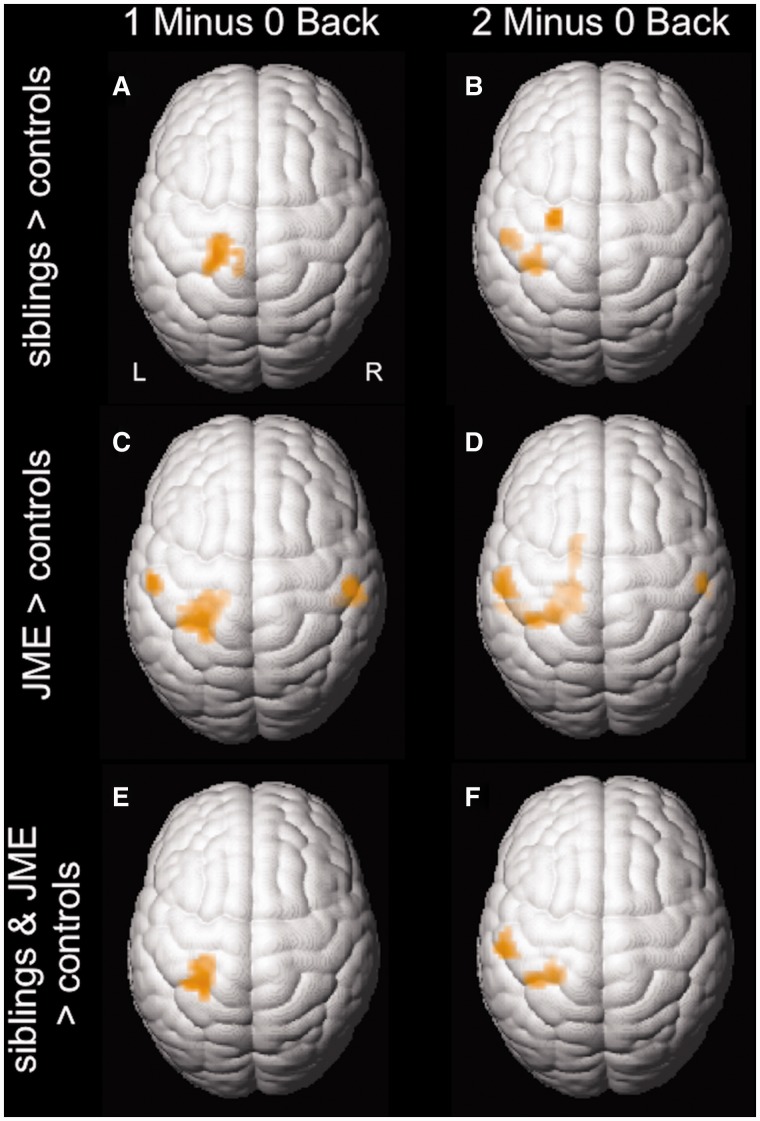
*Post hoc* group comparisons of functional MRI activation patterns after correcting for age. Age was entered as additional nuisance variable into the model. Similar to the results illustrated by Fig. 1, activation patterns in unaffected juvenile myoclonic epilepsy siblings compared to controls (**A** and **B**) showed attenuated deactivation in the motor cortex and supplementary motor area with increasing task demand in the working memory contrasts (*P* < 0.001 uncorrected; 20 voxel threshold extent). At a lower threshold (*P* < 0.005 uncorrected; 20 voxel threshold extent), attenuated deactivation is seen in similar regions in patients with juvenile myoclonic when compared with controls (**C** and **D**). In a conjunction analysis of patients greater than controls and siblings greater than controls for the two working memory contrasts (**E** and **F**), patients and their siblings share significant areas of co-activation in the left motor cortex when compared with controls (conjunction, *P* < 0.005 uncorrected; 20 voxels threshold extent). Maps were inclusively masked for task-dependent deactivation maps of healthy controls. JME = juvenile myoclonic epilepsy.

To control for the effect of age, we performed a *post hoc* group comparison and entered age as an additional regressor of no interest, which did not change the overall results (Fig. 5). In subgroup analyses in patients and siblings, we correlated activation patterns during the ‘2 minus 0 Back’ and ‘1 minus 0 Back’ contrasts with age. This did not show an effect within the region of interest, the left primary motor cortex and supplementary motor area (data not shown).

### Functional connectivity analysis

**Figure 6:**
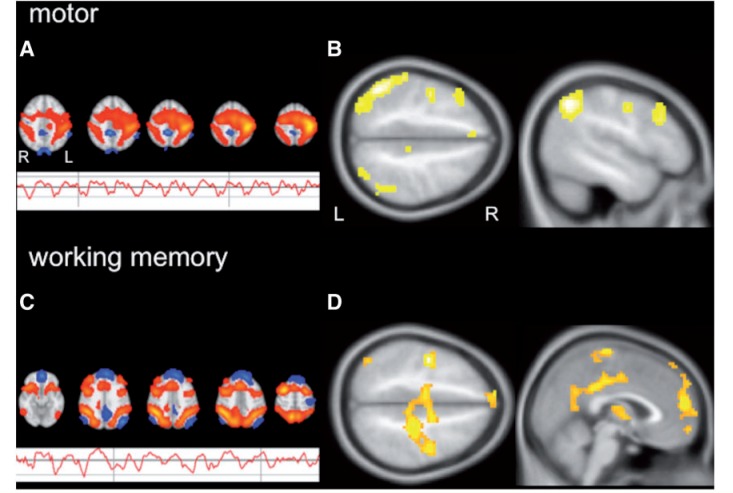
Group independent component analysis and functional connectivity in siblings compared to healthy controls. (**A**) This figure shows the motor network component common to all subjects (FSL figure) and its corresponding group average signal time course during the experiment. The signal time course for the motor component shows constant response amplitude throughout the different task paradigms (0, 1 and 2 Back and rest). (**B**) Group comparison of functional connectivity patterns in siblings and healthy controls are demonstrated for the motor component. Siblings show increased connectivity to fronto-parietal cognitive networks when compared to controls (*P* < 0.005; 20 voxels threshold extent). There were no areas of increased connectivity in controls. (**C**) The working memory network component common to all subjects is demonstrated (FSL figure). Its corresponding group average signal time course is modulated by task demand and shows increased activation with higher cognitive demand during the actual working memory conditions (1 and 2 Back). (**D**) Group comparison of functional connectivity patterns in siblings and healthy controls for the working memory component shows increased connectivity to central motor areas, as well as the medial prefrontal cortex as part of the default mode network (**D**; *P* < 0.001; 20 voxels threshold extent). There were no areas of increased connectivity in controls.

From the 32 independent components identified by independent component analysis, two components of interest were chosen for further group comparisons (Fig. 6): the component located in the left central region and representing the motor response (Fig. 6A) and the component comprising the bilateral prefrontal and parietal working memory network (Fig. 6C).

Compared to controls, juvenile myoclonic epilepsy siblings showed increased functional connectivity of the left motor cortex and supplementary motor area to the dorsolateral prefrontal and superior parietal cortex, which are part of the working memory network (Fig. 6B). Functional connectivity analysis of the working memory component showed increased connectivity to bilateral motor cortices in siblings than controls (Fig. 6D). There were no areas of higher connectivity in controls for these two components.

## Discussion

We detected co-activation of the primary motor cortex and supplementary motor area during a functional MRI working memory task in unaffected siblings of patients with juvenile myoclonic epilepsy, similar to patterns seen in patients with juvenile myoclonic epilepsy (Vollmar *et al.*, 2011). In controls, we observed a relative attenuation of activations in the motor cortices with increasing task demand. In patients with juvenile myoclonic epilepsy and siblings, motor areas remained co-activated with task-positive working memory networks, resulting in increased functional connectivity between the motor system and frontoparietal cognitive networks.

### Abnormal motor-system co-activation and connectivity is an endophenotype of juvenile myoclonic epilepsy

Using a conjunction analysis of working memory activation, we identified common areas of impaired attenuation of task-negative networks within the motor cortex for both patients and siblings. We conclude that motor cortex co-activation is not a consequence of seizures or medications. This supports the hypothesis that there is a heritable component of the disease, and represents an endophenotype of juvenile myoclonic epilepsy, defined as an intermediate phenotype that appears to be more frequently present in non-affected family members than in the general population. As siblings do not suffer from seizures, this finding is clearly not solely an association with the full juvenile myoclonic epilepsy phenotype. However, in view of its regional specificity, our finding is very likely to be related to pathomechanisms of the disease with its particular seizure type, i.e. motor seizures, and fronto-cortical cognitive dysfunction. This is corroborated by studies showing a modulation of motor cortex co-activation by disease severity and treatment (Vollmar *et al.*, 2011). In a recent twin study (Blokland *et al.*, 2011), functional MRI activation patterns during the n-back working memory task have been shown to be significantly heritable and regions of interest identified here, i.e. the precentral gyrus and supplementary motor area, have been among the regions with the highest heritability estimates. Thus, seizures and neurobehavioural comorbidities may share this underlying functional mechanism. Longitudinal studies and imaging studies in recent onset idiopathic generalized epilepsies, as well as juvenile myoclonic epilepsy, identified subcortical and fronto-cortical abnormalities, which relate both to seizures and neurobehavioural comorbidities (Pulsipher *et al.*, 2009; Tosun *et al.*, 2011). Some behavioural studies in idiopathic generalized epilepsies identified cognitive impairment even prior to disease onset (Hermann *et al.*, 2012), suggesting that epilepsy and its comorbidities may reflect different degrees of disease with a shared underlying pathological condition, which may be a genetically determined neurodevelopmental dysfunction (Helmstaedter *et al.*, 2014).

Previous imaging studies of unaffected siblings have been conducted mainly in schizophrenia and autism to control for the effect of disease severity and treatment and to identify potential imaging endophenotypes (Callicott *et al.*, 2003; Spencer *et al.*, 2012; Moran *et al.*, 2013). Like juvenile myoclonic epilepsy, these are considered highly heritable, neurodevelopmental conditions with neurobehavioural characteristics, which extend beyond the cardinal disease features and are frequently found in non-affected relatives. Such endophenotypes are intermediate biological phenotypes associated with the disease in the population, which are more closely related to the genotype than the final phenotype, increasing the yield for identifying susceptibility genes (Callicott *et al.*, 2003). Studying the physiological mechanisms underlying neurobehavioural impairments in unaffected siblings may help to understand biological effects of susceptibility genes (Callicott *et al.*, 2003).

Statistical analyses of the blood oxygen level-dependent contrast at single subject level do not directly reflect a quantitative measure of activation and findings at group level cannot be easily used to quantify activation at a single subject level. In the first instance, this would involve studying large cohorts to establish quantitative normative data of task-related activation. Therefore, it is unlikely that one would be able to conclude from the scan data in one subject whether the trait is present or not in that individual. However in schizophrenia, results from functional MRI group analyses have been used successfully in a probabilistic approach for gene discovery in conjunction with genome-wide association (Potkin *et al.*, 2009), whilst imaging studies in siblings of patients with epilepsy are rare (Scanlon *et al.*, 2013). Analysis of a quantitative imaging trait in affected families, like motor cortex co-activation, may increase the yield of genetic studies for identifying culprit genes for juvenile myoclonic epilepsy, which so far has proven difficult.

A recent transcranial magnetic stimulation study in individuals with generalized and focal epilepsies and their asymptomatic siblings reported cortical hyper-excitability in the asymptomatic siblings compared to healthy controls, which was more prominent in generalized epilepsy syndromes. The cortical excitability profile in asymptomatic siblings was similar to those in patients. Only drug-naïve new-onset patients with juvenile myoclonic epilepsy had a lower motor threshold, i.e. higher excitability, than their asymptomatic siblings (Badawy *et al.*, 2013).

To identify whether motor cortex co-activation is more prominent in patients with juvenile myoclonic epilepsy, we carried out a group comparison of patients with juvenile myoclonic epilepsy and siblings, which did not show an effect. This may be a false negative finding due to the relatively small sample of 11 index patients. An alternative explanation for the lack of a difference could be that motor system co-activation ‘normalized’ with high doses of valproate (Vollmar *et al.*, 2011) and was less prominent in our cohort of 11 patients: all were on medication with 7 of 11 on valproate; six patients were seizure-free and none of the patients reported daily jerks.

To further investigate whether motor cortex co-activation is more prominent in patients than siblings, drug-naïve patients with juvenile myoclonic epilepsy have to be studied (Badawy *et al.*, 2013). In a *post hoc* analysis, our findings survived a further correction for age. Disease onset during adolescence coincides with an important phase of brain development. Normal cortical maturation involves thickening or thinning of grey matter during childhood and adolescence, following different developmental trajectories depending on the cortical region and neural system. Grey matter thinning may be associated with synaptic pruning, apoptosis and ongoing myelination, and has been correlated with cognitive and behavioural development. (Jernigan *et al.*, 2011) Decrease in grey matter first involves primary sensorimotor cortices, then secondary and eventually multimodal cortices during late adolescence, such as the dorsolateral prefrontal cortex (Shaw *et al.*, 2008). However, there is also evidence for continuous developmental changes in primary cortical areas during late adolescence (Giorgio *et al.*, 2010). These crucial processes of cortical brain maturation and functional refinement may be implicated in juvenile myoclonic epilepsy. Mutations in one causative candidate gene, *EFHC1*, have recently been linked to alterations of several neural development steps, including migration, connection formation and apoptosis, the latter potentially leading to maintenance of hyperexcitable neurons (De Nijs *et al.*, 2013). There is some evidence from longitudinal structural imaging studies in children with idiopathic epilepsy compared to controls, describing disrupted patterns of brain development, mainly implicating prefrontal and parietal cortices (Tosun *et al.*, 2011). Therefore, aberrant activation patterns may be more prominent in younger subjects. However, this effect was not seen in a subgroup correlation analysis (Fig. 5). Considering that all of our patients, and most of the siblings, were older than adolescence (patients: age range 22 to 54 years; siblings: 18 to 65 years), this may be a false negative finding and a potential age effect should be explored in future, preferably with recent-onset cohorts.

### Abnormal functional MRI activation patterns are markers of dysfunctional traits

Motor system co-activation appears to be not only a disease marker, but is related to cortical network dysfunction. In our previous studies, we suggested that motor cortex co-activation with functional hyper-connectivity and increased microstructural connectivity between the prefrontal cognitive cortex (presupplementary motor area) and motor system is a potential underlying mechanism of cognitively triggered jerks and frontal lobe impairment in juvenile myoclonic epilepsy (Vollmar *et al.*, 2011). Connectivity between the presupplementary motor area region and the frontopolar cortex was reduced, providing an explanation for impaired frontal lobe functions in juvenile myoclonic epilepsy. In addition, thalamic inhibition of the supplementary motor area and premotor cortex has been shown to be decreased in association with reduced structural connectivity within thalamo-cortical motor control circuits, which leads to alteration of task-modulated functional connectivity with subsequent impairment of frontal lobe functions (O'Muircheartaigh *et al.*, 2012). The effect appeared more prominent in patients with persisting seizures. Likewise, impairment in experience-related learning and impulsive decision-making have been directly related to increased supplementary motor area activation in treatment of patients with refractory juvenile myoclonic epilepsy (Wandschneider *et al.*, 2013).

Comparative studies of patients and controls, however, have failed to disentangle whether structural and functional changes are part of disease-underlying mechanisms or a consequence of seizures and/or treatment. In our current study, we control for the impact of seizures and medication by studying unaffected siblings. Similar findings in affected and unaffected family members support the contention that altered structural and functional cortico-cortical connectivity is part of the genetically determined disease-underlying mechanisms. To compare our current with previous findings in patients with juvenile myoclonic epilepsy (Vollmar *et al.*, 2011), task-related, but not resting state functional connectivity was assessed. In a recent meta-analysis of >7000 functional maps, the main explicit activation networks were identified and compared to those identified in 36 subjects during resting state functional MRI (Smith *et al.*, 2009). Major co-varying network components of the task-related analysis were very similar to those in the resting brain (Laird *et al.*, 2011). A task-related functional connectivity analysis approach appears appropriate in juvenile myoclonic epilepsy, as symptoms become more apparent during certain activities or with increasing cognitive demand.

As in patients, unaffected siblings show increased functional connectivity between working memory networks and motor systems and vice versa. Siblings demonstrate this imaging trait, but they do not experience seizures, which indicates that additional environmental and/or genetic factors are necessary to develop the full juvenile myoclonic epilepsy phenotype. On the other hand, motor cortex co-activation and hyper-connectivity may not only be a genetic marker but may be associated with disease traits in siblings. Previous studies have shown subtle frontal lobe impairment in unaffected juvenile myoclonic epilepsy siblings (Levav *et al.*, 2002; Wandschneider *et al.*, 2010), especially when performing a cognitively challenging task that required integration of several frontal lobe functions (Wandschneider *et al.*, 2010). In the current study, siblings performed less well on the highly demanding functional MRI working memory task, although they did equally well on the standardized neuropsychological test battery. Hence altered task-related functional connectivity between motor and cognitive networks demonstrated in this study may be responsible for subtle cognitive impairments in siblings that are similar to those in patients.

### Impaired task-related deactivation of motor systems

Motor cortex co-activation in siblings and patients compared to controls was due to attenuated deactivation of the motor systems. Group effects of task-related deactivations showed deactivation of areas of the motor cortex in controls, but to a lesser degree in siblings (Fig. 2). In patients with juvenile myoclonic epilepsy, an independent component analysis previously identified a ‘modulated motor’ component during the n-back working memory task, which demonstrated that, similarly to the working memory component here (Fig. 6), the motor component was modulated with increasing working memory task demand (Vollmar *et al.*, 2011). In the current cohort, functional connectivity in siblings was increased between working memory networks and areas, which were deactivated in controls, i.e. motor cortices and the medial prefrontal cortex as part of the default mode network. Due to increased functional coupling of cognitive and motor networks in patients with juvenile myoclonic epilepsy and their unaffected siblings, functional segregation of motor areas from task-active cognitive networks and their deactivation during a highly demanding working memory task may be impaired, which may account for the poorer performance in siblings during the functional MRI working memory task in this study.

### Limitations

Interictal epileptic discharges have been reported in up to 27% of unaffected siblings of patients with juvenile myoclonic epilepsy (Atakli *et al.*, 1999) and may therefore be also present in our sibling cohort. A recent sibling study (Iqbal *et al.*, 2009) controlling for interictal epileptic activity by performing video EEG recordings before and during neuropsychological assessment reported subtle cognitive impairment in siblings and patients independently of interictal epileptic discharges. Given the low sensitivity to detect interictal epileptic discharges routine EEGs were not performed in siblings for this study. We also postulate that functional MRI is a far more sensitive tool to detect subtle neuronal dysfunction in clinically unaffected individuals and this has already been achieved in previous cognitive functional MRI studies despite the absence of impairment on routine neuropsychological tests (Vollmar *et al.*, 2011; Spencer *et al.*, 2012).

One of the siblings had experienced two seizures more than 20 years before study participation. However, these seizures were clearly provoked. There was no evidence of further unprovoked seizures and no anti-epileptic medication had been taken. As affected participants were defined as individuals with recurrent unprovoked seizures, this participant was not excluded from the study. Excluding this data set from the analysis did not alter the overall results.

## Conclusion

Attenuated deactivation of the motor system and increased functional connectivity between fronto-parietal cognitive networks and the motor cortex occurred both in patients with juvenile myoclonic epilepsy and their unaffected siblings during a functional MRI working memory task. Our findings most likely reflect an imaging endophenotype of juvenile myoclonic epilepsy, representing the shared underlying genetic risk of juvenile myoclonic epilepsy in both disease-affected and -unaffected siblings, and therefore providing a potential biomarker for future genetic imaging studies.

## Funding

We are grateful to the Wolfson Trust and the Epilepsy Society for supporting the Epilepsy Society MRI scanner. This research was supported by the National Institute for Health Research University College London Hospitals Biomedical Research Centre. B. Wandschneider was funded by a fellowship of the German Research Foundation (Deutsche Forschungsgemeinschaft; WA 3135/1-1). The study was funded through the Wellcome Trust (Project Grant No 079474) and a medical research grant from the Henry Smith Charity (ref 20133416).
